# Atomic Force Microscopy Reveals a Morphological Differentiation of *Chromobacterium violaceum* Cells Associated with Biofilm Development and Directed by N-Hexanoyl-L-Homoserine Lactone

**DOI:** 10.1371/journal.pone.0103741

**Published:** 2014-08-11

**Authors:** Anara A. Kamaeva, Alexey S. Vasilchenko, Dmitry G. Deryabin

**Affiliations:** 1 Department of Microbiology, Orenburg State University, Orenburg, Russia; 2 Laboratory of Disbiosis, Institute of Cellular and Intracellular Symbiosis, RAS, Orenburg, Russia; 3 RSE «Republican Collection of Microorganisms», Astana, Republic of Kazakhstan; LAAS-CNRS, France

## Abstract

*Chromobacterium violaceum* abounds in soil and water ecosystems in tropical and subtropical regions and occasionally causes severe and often fatal human and animal infections. The quorum sensing (QS) system and biofilm formation are essential for *C. violaceum's* adaptability and pathogenicity, however, their interrelation is still unknown. *C. violaceum's* cell and biofilm morphology were examined by atomic force microscopy (AFM) in comparison with growth rates, QS-dependent violacein biosynthesis and biofilm biomass quantification. To evaluate QS regulation of these processes, the wild-type strain *C. violaceum* ATCC 31532 and its mini-Tn5 mutant *C. violaceum* NCTC 13274, cultivated with and without the QS autoinducer N-hexanoyl-L-homoserine lactone (C_6_-HSL), were used. We report for the first time the unusual morphological differentiation of *C. violaceum* cells, associated with biofilm development and directed by the QS autoinducer. AFM revealed numerous invaginations of the external cytoplasmic membrane of wild-type cells, which were repressed in the mutant strain and restored by exogenous C_6_-HSL. With increasing bacterial growth, polymer matrix extrusions formed in place of invaginations, whereas mutant cells were covered with a diffusely distributed extracellular substance. Thus, quorum sensing in *C. violaceum* involves a morphological differentiation that organises biofilm formation and leads to a highly differentiated matrix structure.

## Introduction

*Chromobacterium violaceum* is a Gram-negative, facultative anaerobic, non-sporing beta-proteobacterium that is abundant in soil and water ecosystems in tropical and subtropical regions [1]–[3]. As a typical saprophyte, this bacterium occasionally becomes an aggressive opportunistic pathogen causing severe and often fatal human and animal infections [4]–[8].

The remarkable versatility and adaptability of *C. violaceum* have been explained by complete genome sequencing of this bacterium [9]; this revealed 4,431 open reading frames (ORFs) associated with energy generation, transport, signal transduction, cell motility, secretion, and secondary metabolism [10], [11], such as is important for mammalian pathogenicity proteins [12]. Finally, a large number of ORFs related to the regulation of gene expression were also found, and widespread utilisation of inducible quorum sensing systems to detect and respond to changes in cell population density have been demonstrated.

*C. violaceum* uses a LuxIR-type quorum sensing system [13]–[15] in which CviI is an N-hexanoyl-L-homoserine lactone (C_6_-HSL) synthase, and CviR is a cytoplasmic receptor protein (DNA-binding transcription factor) that activates gene expression following binding to the diffusible autoinducer C_6_-HSL [16]. Among a number of quorum sensing-controlled genes, the most well known is the *vioABCDE*-operon [17]. Expressed at high cell density, VioA – E proteins carry out the enzymatic oxidation and coupling of two molecules of tryptophan to give a rearranged pyrrolidone-containing scaffold in the final purple pigment violacein [18].

Another important activity of *C. violaceum* is self-produced polymer matrix (biofilm) formation [19] that structures the bacterial consortia and is linked to virulence through resistance to antibiotics, disinfectives and phagocytosis. Like several pathogenic bacteria, *C. violaceum* has the hms*HFR*-CV2940 operon, whose products synthesise the *N*-acetyl-D-glucosamine monomer and polymerise it in the biofilm exopolysaccharide [20]. Furthermore, bacteria communicate with each other in biofilms by diffusible autoinducers, though the role of cell-to-cell signalling in *C. violaceum* biofilm development is not characterised.

Using *in vivo* data generated from a library of point mutations in a CviR-regulated promoter, Stauff and Bassler [16] scanned the *C. violaceum* genome to predict quorum sensing-regulated CviR binding sites. It has been confirmed that CviR controls *cvi*I (see below) and chitinase [16], [21], and it also participates in the expression of transcriptional regulators, guanine deaminase, and the type VI secretion-related gene. However, the DNA motif bound by CviR is absent in the *hmsHFR*-CV2940 operon. Thus, a canonical quorum-sensing control of *C. violaceum* biofilm formation is still unknown.

These inconsistent data have led us to investigate biofilm formation by a wild-type *C. violaceum* strain and its mini-Tn5 mutant, the latter of which is deficient in autoinducer production but retains the QS mechanisms in response to exogenous C_6_-HSL. Using atomic force microscopy (AFM), we have revealed for the first time a morphological differentiation of *C. violaceum* cells associated with biofilm formation and directed by C_6_-HSL.

## Materials and Methods

### Bacterial strains and culture conditions

The wild-type strain *C. violaceum* ATCC 31532 was acquired from the American Type Culture Collection (LGC Standarts, UK), and *C. violaceum* NCTC 13274 from the National Collection of Type Cultures (Health Protection Agency, UK). The mini-Tn5 mutant of the wild-type strain has an insertion in the *cvi*I gene, and it is deficient in the production of its own autoinducer but retains the ability to respond on N-hexanoyl-L-homoserine lactone and a variety of other (C_4_–C_8_) short-chain HSLs [22], [23]. Therefore, *C. violaceum* NCTC 13274 is colourless, but produces a characteristic purple pigment violacein by incubation with C_6_-HSL, which is widely used as an indicator in the study of quorum sensing mechanisms.

*C. violaceum* ATCC 31532 was grown in 3 ml of Luria–Bertani (LB) broth (AppliChem GmbH, Germany) with 10 mg/ml glucose at 30°C. *C. violaceum* NCTC 13274 was cultivated in the same conditions with and without 0.1 µM of C_6_-HSL obtained from the Cayman Chemical Company (Ann Arbor, USA). At 24, 48 and 72 h intervals, the biomass from single vials was collected, and bacterial growth was observed by measuring the absorbance at 450 nm (OD_450_) using a Stat Fax 303 6VIS microstrip reader (Awareness Technology Inc., (Palm, USA).

### Violacein extraction

Bacterial cells were harvested by centrifugation at 10,000× g for 5 min (MiniSpin, Eppendorf, 1 ml), and the supernatant was removed. To extract violacein, 200 µl of 95% ethanol was added and samples were vortexed at room temperature for 5 min. After centrifugation (10,000× g for 5 min), the upper phase containing violacein was collected, and the culture was re-extracted with a second volume of ethanol. The extracts were combined, and the relative concentrations of violacein were measured using a microstrip reader at 575 nm (OD_575_) [24].

### Crystal violet binding assay

The biofilm biomass quantification of *C. violaceum* was determined by crystal violet staining [25]. Because the dye and violacein have very similar wavelength spectra (maximal absorbance 540 and 575 nm for crystal violet and violacein, respectively), the protocol for biofilm quantification was slightly modified, and ethanol extracted colourless air-dryed biomass (see above) was used for the binding assay. A total of 200 µl of an aqueous solution of crystal violet (0.1%) was added to each well and incubated at room temperature. The staining solution was removed, and probes were washed twice (10,000× g for 5 min) with 200 µl of deionised water to remove excess dye. The dye that was strongly bounded to the biofilm was solubilised with 200 µl of 95% ethanol for 5 min, washed and re-extracted with a second volume of ethanol. Relative quantitative determinations of crystal violet were measured in combined samples by reading the optical density at 540 nm (OD_540_) [26].

### Atomic force microscopy

For AFM, the planktonic and biofilm-forming *C. violaceum* were individually imaged. Planktonic bacteria were pipetted out by touching the top of the tips to the corner of each well, washing from the cultivation media (3,000× g for 5 min), re-suspending in deionised water; then droplets of 15-µl bacterial suspensions were applied to a freshly cleaved mica surface (5×5 mm). Remaining biofilms were carefully transferred under sterile conditions to a mica surface immediately. Samples were dried with controlled 93% humidity according to [27].

The observations was obtained with an atomic force microscope SMM-2000 (JSC “Proton-MIET Plant”, Russia) in contact mode in air. The instrument was equipped with silicon nitride cantilever MSCT-AUNM (Veeco Instruments Inc., USA) with a pyramidal (V-shaped) tip with a typical radius of ∼10 nm and a spring constant of 0.01 N/m.

During AFM imaging, the root-mean-square roughness (R_rms_ – the standard deviation of the Z values) for the height images were conducted over 5- µm by 5- µm areas on different biofilm surfaces and was calculated using SMM-2000 software. The images were flattened and plane fitted prior to analysis.

### Statistical analysis

All the experiments were performed at least three times in triplicate. The data are reported as means ± standard deviation. Two-tailed *t*-test statistics were used at significance levels of 0.05 and 0.01.

## Results

### Growth, violacein production and biofilm formation by wild-type strain *C. violaceum* ATCC 31532

**Figure 1:**
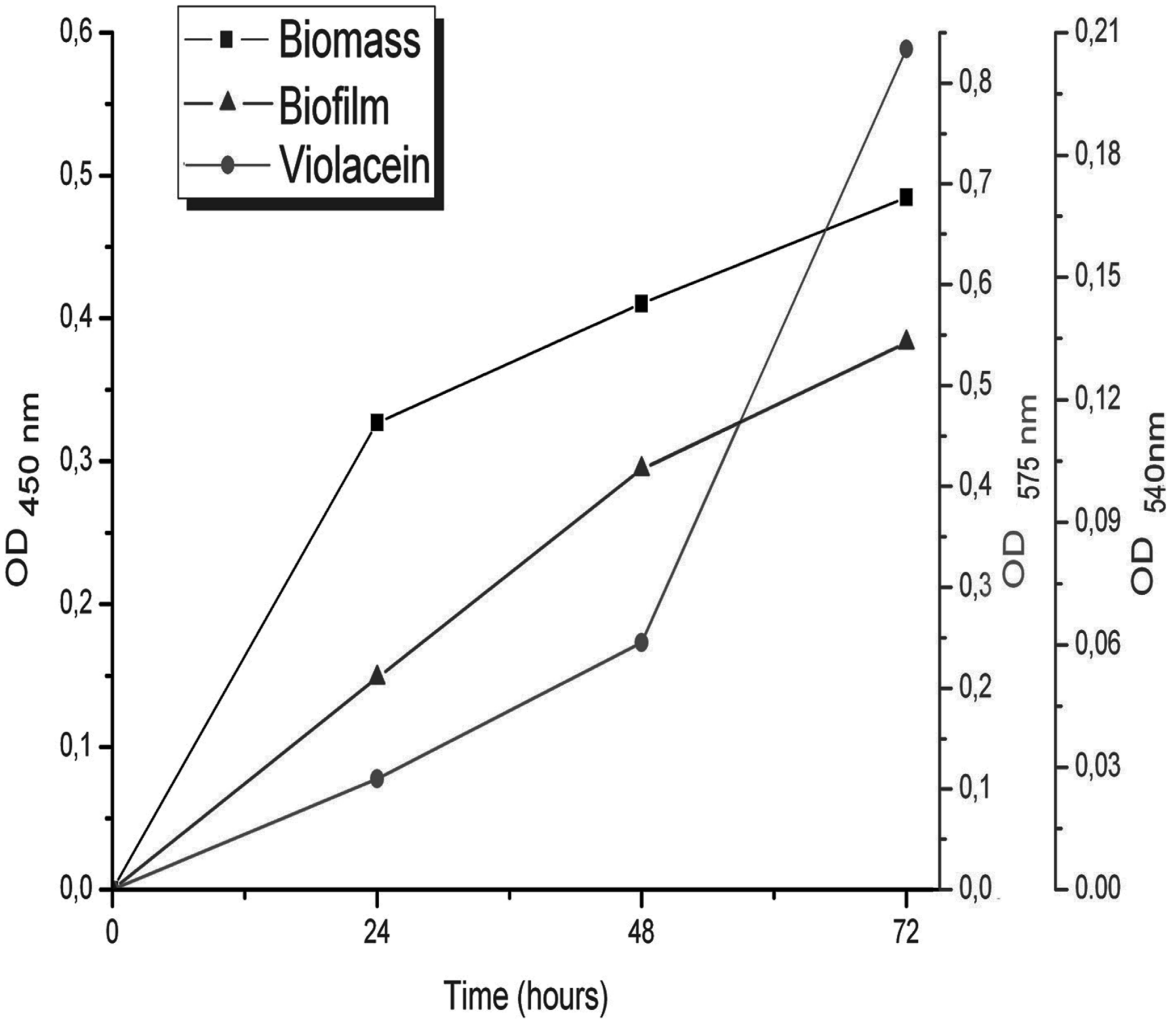
Time course (a) of the cell density (OD _450_) for violacein (OD _575_) and biofilm (OD _540_) production by wild-type strain *C. violaceum* ATCC 31532, growing in LB broth for 24, 48 and 72 h.

In order to evaluate if violacein production and biofilm formation were dependent on cell density, *C. violaceum* ATCC 31532 was grown in LB broth for 24, 48 and 72 h (Fig. 1). This experiment showed a typical growth (OD_450_) curve with maximal progression in 24 h followed by a gradual increase to the late stationary phase. Production of violacein was detected at 24 h, but the most growth of characteristic OD_575_ was established at high cell density between 48 and 72 h where this value increased 3.4-fold. On the other hand, the biofilm production was quite similar to the growth rate, and the OD_540_ after crystal violet staining increased proportionally to an OD_450_. Thus, violacein and biofilm in the wild-type strain *C. violaceum* ATCC 31532 differentially corresponded to cell density and only the first developed in a fashion similar to a quorum-dependent parameter.

### AFM imaging of wild-type strain *C. violaceum* ATCC 31532 cells and biofilm

**Figure 2:**
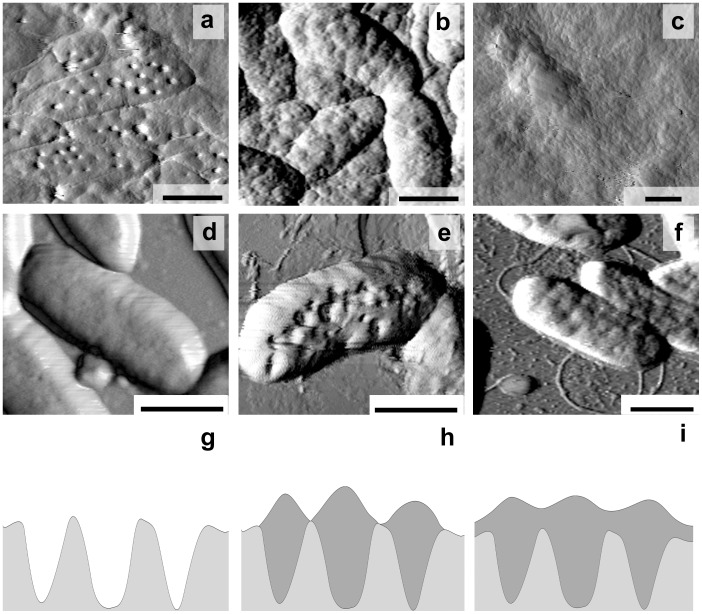
AFM images (a–f) and schematic model of extracellular matrix secretion (g–i) by wild-type strain *C. violaceum* ATCC 31532, growing for 24 (a, d, g), 48 (b, e, h) and 72 h (c, f, i) in biofilmic (a–c; g–i) and planktonic (d–f) conditions. Scale bar – 1 µm.

The planktonic and biofilm-forming bacteria growing for 24, 48 and 72 h were immobilised on a mica surface to observe the cells' general morphology and detailed surface topography (Fig. 2).

**Figure 3:**
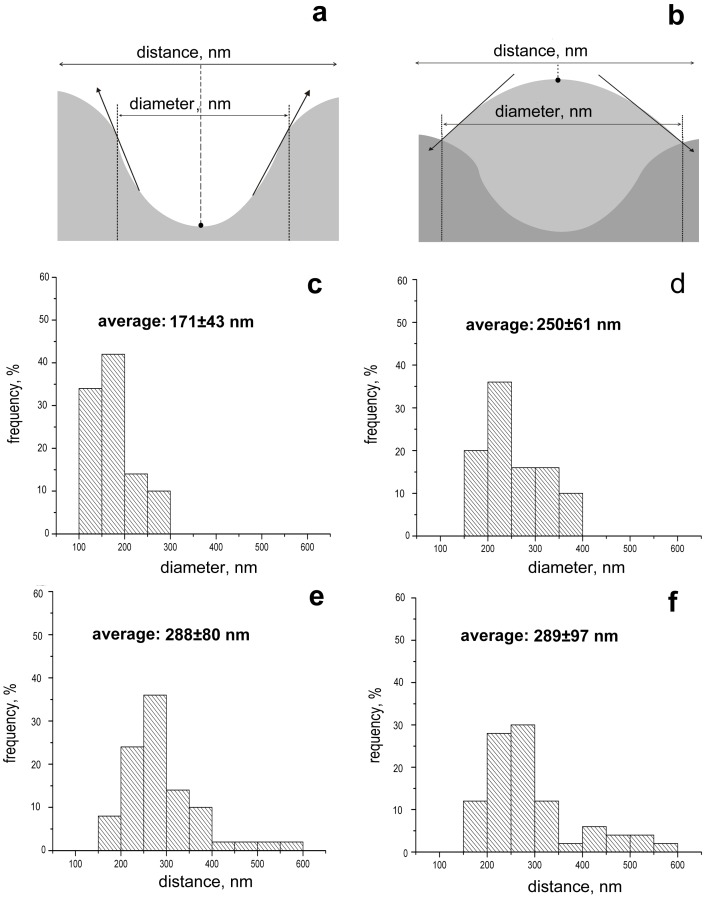
Dimensional specifications of morphological features on the surface of cells (invagination and extrusion) were assessed on the basis of AFM profile data carried out through two adjoined invaginations or extrusion. Distance was defined as a spacing between points located: in center of two adjoined invaginations (a) or on the top of two adjoined extrusions (b). Diameter of invaginations and extrusions is defined as a distance between two points that are located on two crossing lines (solid line and dotted line). Precise morphometry of *C. violaceum* ATCC 31532 cells surfaces (in biofilm conditions), revealed: diameters of invaginations (c) and extrusions (d); distances between centres of two adjoined invaginations (e) and two adjoined extrusions (f) (histograms and average value).

The 24-h individual *C. violaceum* cells had a characteristic elongated shape, and based on AFM, the cells were 2.45±0.31 µm length, 0.85±0.09 µm wide and 0.34±0.07 µm high and without distinctions between planktonic and biofilm-forming bacteria. However, the surface topography of these cells was significantly different, and unusual invaginations (about 30–40 per cell taking into consideration 50% cell surface visibility) of the external membrane in biofilm-forming bacteria were observed (fig. 2 a). Based on AFM profiles, the invaginations had a 171±43 nm diameter (Fig. 3 c).and a 31.1±7.5 nm depth, whereas the distance from their centres were nearly 288±80 nm (Fig. 3 e).

At 48 h invaginations similar to these observed with biofilm-forming cells at 24 h (fig. 2 a) are observed on planktonic cells (Fig. 2 e), while biofilm-forming bacteria became essentially different at the same time (Fig. 2 b). High-resolution AFM images (Fig. 2 b) of biofilm-forming bacterial cells surfaces showed that the bacteria were covered with granular structures, and hill-like extrusions had appeared.

The extrusions' average diameter measured by AFM profiles was 250±61 nm (Fig. 3 d), and the average distance from their tops was 289±97 nm (Fig. 3 f). The arrangement and distance centre-to-centre of the 24-h invaginations and 48-h extrusions (*P* = 0.4673) have assured us of their spatial interrelation, that the extrusions formed in place of the previous invaginations. On the other hand, comparison of *C. violaceum* growth and AFM images has allowed us to explain processes occurring by biofilm's matrix production.

At 72 h, AFM revealed the granulated biofilm surface and contours of biofilm-forming bacterial cells (Fig. 2 c). At the same time, the planktonic bacteria changed and also developed extrusions on their surface (Fig. 2 f). In addition, flagella (approx. 20 nm in diameter) and some other thinner fimbrial structures were detected in planktonic samples.

Thus, using AFM, we report for the first time the morphological differentiation of C. violaceum ATCC 31532 cells increasing with cultivation time and preliminarily developed in biofilm-forming cells, whereas similar changes in the planktonic cells have developed late. However, the quorum-sensing control of *C. violaceum* cell differentiation and biofilm formation remains unclear.

### Growth, violacein production and biofilm formation by the mutant strain *C. violaceum* NCTC 13274

**Figure 4:**
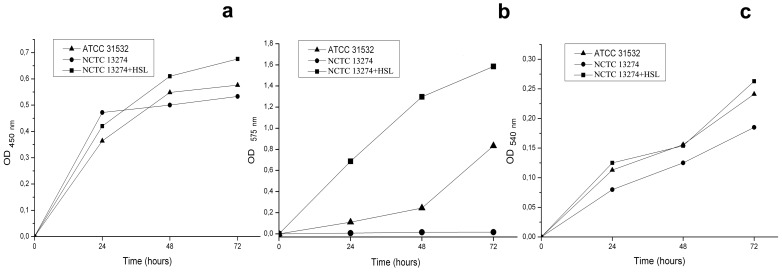
Time course of cell density (a), for violacein production (b) and biofilm formation (c) by wild-type strain *C. violaceum* ATCC 31532 in comparison with mutant strain *C. violaceum* NCTC 13274, growing in LB broth without and with 0.1 µM C_6_-HSL.

In order to evaluate the effects of quorum sensing on biofilm formation, we investigated the mini-Tn5 mutant strain *C. violaceum* NCTC 13274 cultivated without and with the natural autoinducer N-hexanoyl-L-homoserine lactone (Fig. 4).

When *C. violaceum* NCTC 13274 were grown for 24, 48 and 72 h, both in LB broth and in LB broth with 0.1 µM C_6_-HSL, violacein production differed cardinally (“no” or “yes”), whereas growth rates and growth curves were comparable. After 24 h of incubation, the OD_450_ value of a colourless culture, grown in the absence of autoinducer, was approximately 11.0% higher than that in the presence of C_6_-HSL, and approximately 22.9% higher than that of the wild-type strain *C. violaceum* ATCC 31532. In our opinion, this could be due to energy savings on violacein biosynthesis in comparison with HSL-deficient strain, but 48 and 72 h growth rates of the colourless cultures became less than those of pigmented ones. On the other hand, violacein production by *C. violaceum* NCTC 13274 incubated with C_6_-HSL was significantly higher than that of the wild-type strain *C. violaceum* ATCC 31532, and the characteristic OD_575_ in comparison with a wild strain increased 4.2-fold in 24 h, 5.3-fold in 48 h and 1.9-fold in 72 h, respectively. We suspect it was because exogenous C_6_-HSL was available at the growth start, and *C. violaceum* NCTC 13274 developed violacein biosynthesis irrespective of the cell density: growth curves for wild-type and mutant strains were very similar.

Contrary to the qualitative distinctions of violacein production, biofilm formation by *C. violaceum* NCTC 13274 with and without C_6_-HSL only differed quantitatively. These processes were quite similar in *C. violaceum* NCTC 13274 growing with C_6_-HSL and in the wild-type strain *C. violaceum* ATCC 31532, while staining of colourless culture using the crystal violet revealed only 64.0–70.3% biofilm biomass in comparison with the pigmented culture.

In brief, if the mini-Tn5 insertion in the *cvi*I gene led to the block in violacein biosynthesis, and the exogenous autoinducer restored and strengthened it considerably, then biofilm formation decreased quantitatively, and the autoinducer only restored it. Thus, violacein production developed like a quorum-dependent parameter, whereas biofilm formation occurred in a non-canonical fashion, i.e. biofilm formation occurred without direct control by a QS-inducer, but was associated with luxR/luxI genes.

### AFM imaging of *C. violaceum* NCTC 13274 cells and comparative study of biofilms' surfaces

**Figure 5:**
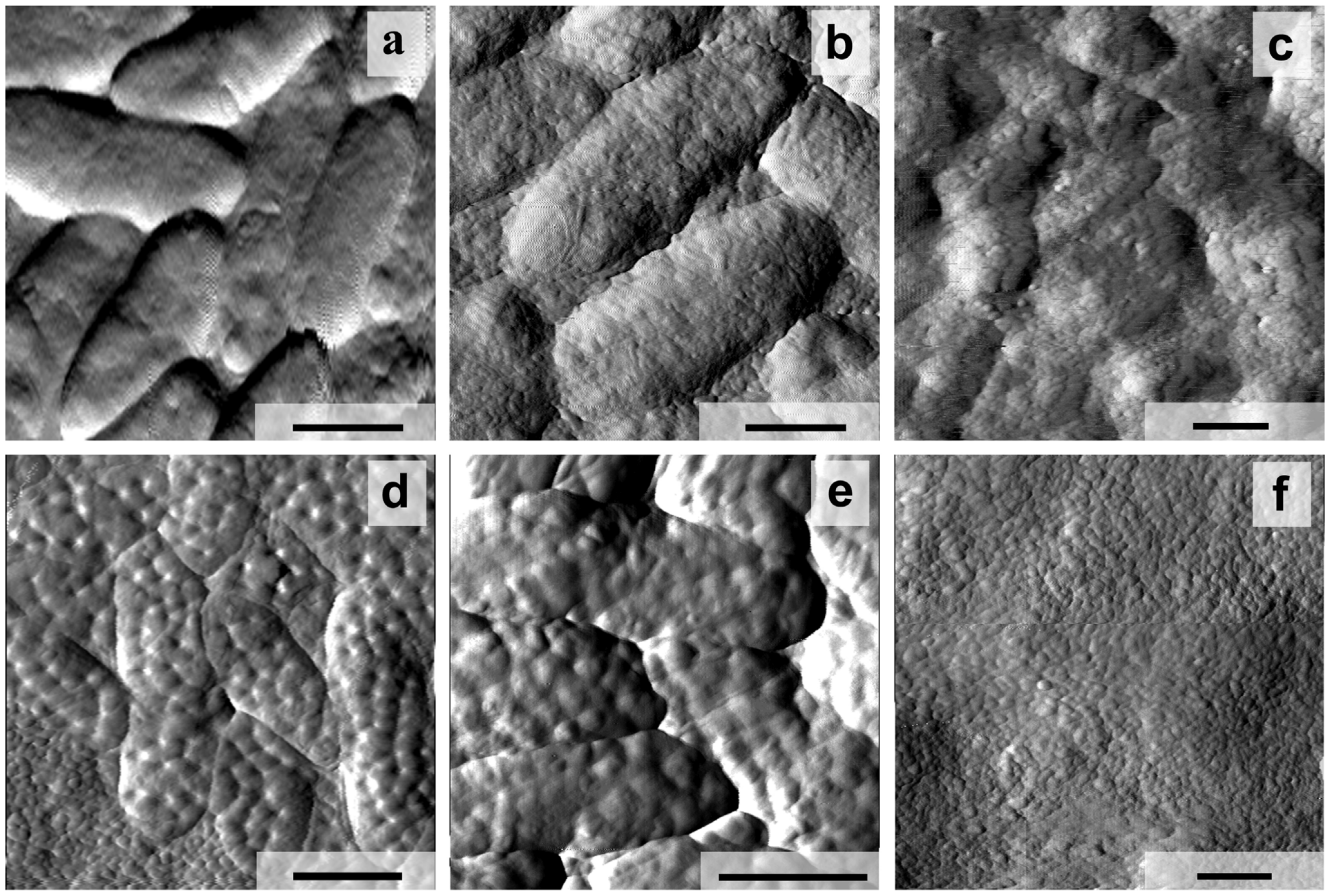
AFM images of biofilm-forming *C. violaceum* NCTC 13274, growing in LB broth without (a–c) and with 0.1 µM C_6_-HSL (d–f) for 24 (a, d), 48 (b, e) and 72 h (c, f). Scale bar – 1 µm.

To confirm or reject the quorum-sensing control of biofilm formation, *C. violaceum* NCTC 13274 cells cultivated with and without C_6_-HSL were imaged by the AFM technique (Fig. 5 and Fig. S1).

Surprisingly, 24-h biofilm-forming cells growing without autoinducer displayed a smooth surface (Fig. 5 a), whereas cells in the presence of C_6_-HSL had numerous invaginations (Fig. 5 d). The topography of invaginations of *C. violaceum* NCTC 13274 cells growing with autoinducer was very similar in depth and diameter to that in the the wild-type strain *C. violaceum* ATCC 31532. At the same time, there were more invaginations (about 50–80 per cell taking into consideration 50% cell surface visibility), the distance from their centres was less (155±21 nm), and the arrangement was more ordered. These data strongly suggest that *C. violaceum* cells differentiate in a quorum-dependent manner that is naturally developed in culture growth, and which is cancelled without autoinducer and resumes with exogenous C_6_-HSL.

At 48 h, AFM revealed the numerous extrusions on the *C. violaceum* NCTC 13274 cell's surfaces growing with C_6_-HSL. This extrusions were like wild-type biofilm-forming cells (see above), and the number per cell, such as distance from extrusion tops, was very similar to the 24-h invaginations.

On the other hand, *C. violaceum* NCTC 13274 cultivated without C_6_-HSL had no invaginations or extrusions, while high-resolution AFM revealed that the bacterial cell surfaces had been covered with a diffusely distributed extracellular substance.

The study of the *C. violaceum* NCTC 13274 planktonic cells without C_6_-HSL also shown their smooth surface (Fig. S1 a–c) but addition of autoinducer restored their morphological differentiation (Fig. S1 d–f), however it was less pronounced compared with biofilm-forming cells.

Because AFM is capable of surface morphology imaging with nanometre resolution, we used this technique for comparative study of mature 72-h biofilms formed by the wild-type strain *C. violaceum* ATCC 31532 and mini-Tn5 mutant strain *C. violaceum* NCTC 13274 cultivated with and without C_6_-HSL.

**Figure 6:**
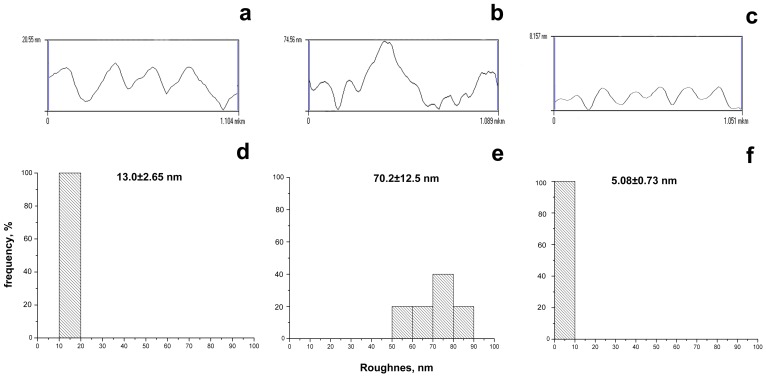
Cross sections (a–c), where x-axis is a heights of relief points and y-axis is a cross sections distance; and roughness values (d–f) of biofilm surfaces, produced by wild-type strain *C. violaceum* ATCC 31532 (a, d) and mutant strain *C. violaceum* NCTC 13274, growing without (b, e) and with 0.1 µM C_6_-HSL (c, f).

Figure 6 shows that *C. violaceum* ATCC 31532 produced relatively smooth biofilm consisting of tightly associated polymer blocks, and the average surface roughness was only 13.0±2.65 nm.

Noticeably, deficiency in autoinducer production *C. violaceum* NCTC 13274 exhibited a more irregular biofilm surface structure with a average roughness of 70.2±12.5 nm (*P* = 0.0007) that was probably caused by breakage of the quorum-dependent secretion machinery. At last, topography AFM images of biofilms formed by *C. violaceum* NCTC 13274 cultivated with exogenous C_6_-HSL shows that there is no significant difference between roughness in the wild-type and mutant strains (average value 5.08±0.73 nm; *P* = 0.0018).

Thus, quorum sensing in C. violaceum does not control intracellular exopolysaccharide biosynthesis but probably involves the secretion of these molecules as extracell biofilm structure development, and AFM shows unusual bacterial cell morphological differentiation, which is associated with biofilm development and is directed by N-hexanoyl-L-homoserine lactone.

## Discussion

Biofilm formation is the result of a complex process that involves mono- and polysaccharide biosynthesis, its transport through a microbial cell wall and a subsequent arrangement of structure and architecture of the biofilm matrix [28]–[30]. Among diverse regulatory mechanisms postulated for biofilm formation, the significant system is quorum sensing that involves production, release and detection of small signalling molecules (autoinducers). This allows microbial cells to activate target gene expression in a cell density-dependent manner, including genes involved in biofilm development and maturation [31]–[34]. However, because these mechanisms vary between different bacterial species, the role of quorum sensing in biofilm formation cannot be described in general terms [35].

Several independent research groups have demonstrated that quorum sensing is essential for biofilm formation by *Pseudomonas aeruginosa* to express virulence factors. This microorganism has hierarchically arranged quorum sensing, with the LasIR on top, controlling the RhlIR, and the additional PQS/HHQ/MvfR systems [36], [37]. In particular, *lasI* mutants that did not produce the specific autoinducer 3-oxo-C12-HSL formed thinner and undifferentiated biofilms than in wild-type, and mutant biofilms appeared normal when *P. aeruginosa* grew in the presence of a synthetic signalling molecule [38]. However, in some recent studies, there was no apparent difference in the biofilms formed by the quorum-sensing mutants and the wild-type strains [39], [40]. Finally, the detailed *P. aeruginosa* virulence regulatory network did not show the direct quorum-sensing control of biofilm formation, delocalised on cell regulatory pathways under the key BfiSR, MifR and BfmSR activators [41].

*C. violaceum* has a simple unicomponent quorum-sensing system [13]–[15] where the *cviI* gene encodes the autoinducer (C_6_-HSL) synthase, and *cviR* encodes a cytoplasmic receptor protein that binds C_6_-HSL and activates expression of target genes [16]. However quorum-sensing control of biofilm formation by *C. violaceum* is not clear because specific CviR binding sites are absent in the exopolysaccharide biosynthesis operon [16].

Here, we describe the use of atomic force microscopy (AFM) combined with classical bacteriological techniques to reveal and quantify *C. violaceum* quorum sensing and biofilm formation. In these experiments, wild-type and *cviI* mutant bacteria allow to estimate both phenomena in violacein production and crystal violet staining tests, respectively. In turn, the use of AFM allows visualisation of cell morphology, matrix distribution and biofilm formation that can be carried out in their native state and under physiological conditions. Thus, AFM requires minimal sample preparation and creates 3D images with nanometer or sub-nanometer resolutions.

Our basic conclusion was that dependence between quorum sensing and biofilm formation in *C. violaceum* is not typical for luxI/luxR manner. The evidence for this comparison was as follows: (i) different dynamics of strictly quorum-dependent violacein and biofilms production during wild-type strain *C. violaceum* ATCC 31532 growth; (ii) the absolute block of violacein biosynthesis in the *C. violaceum* NCTC 13274 insertion mutant, whereas biofilm production is few lower than in cases of wild type and mutant strain with inducer; (iii) the considerable strengthening in *C. violaceum* NCTC 13274 violacein biosynthesis by exogenous C_6_-HSL in comparision with wild-type strain *C. violaceum* ATCC 31532, whereas biofilm formation was slightly increased to reach its wild type value only. Thus, like *P. aeruginosa* [39], [40], the *C. violaceum* wild-type strain and quorum-sensing mutants did not show apparent quantitative differences in the biofilm formation.

Surprisingly, AFM revealed an unusual *C. violaceum* cell morphological differentiation, associated with biofilm development and directed by a quorum-sensing autoinducer. AFM revealed numerous invaginations of the external cytoplasmic membrane of wild-type cells, which was repressed in the mutant strain and restored by exogenous C_6_-HSL. With increasing bacterial growth, the extrusions composed apparently from biofilm matrix were formed in place of invaginations, whereas mutant cells were covered with a diffusely distributed extracellular substance. Finally, quorum sensing-controlled C. violaceum formed a fine biofilm structure that differed from non-QS-controlled *C. violaceum*. In summary, our results suggest that quorum sensing controls biofilm quality but not quantity, and without this control bacteria produce undifferentiated biofilms, unlike wild-type biofilm. Therefore, authors assume that biofilm under QS-control become strong and steady against mechanical and chemical stress as shown peviously in [38].

**Figure 7:**
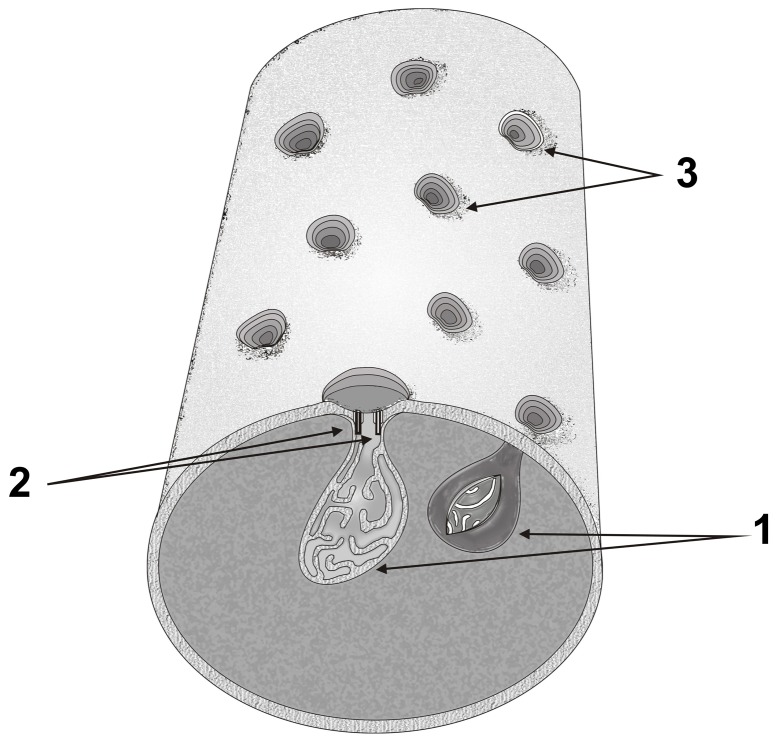
Predicted model of topological spatial relations between mesosomal-like structures (1), type VI secretion proteins (2) and membrane invaginations (3) in C_6_-HSL induced *Chromobacterium violaceum* cells. This art was generated by Corel Draw 13 (Corel Corp.) and Adobe Photoshop CS 5 (Adobe Sys. Inc.).

As part of our experimental result, we were interested in determining the mechanisms of morphological differentiation and biofilm matrix development controlled by quorum sensing. The present study by AFM confirmed the existence of *C. violaceum's* cytoplasmic membrane invaginations as revealed by transmission electron microscopy previously [42]. On this basis, we propose the association of the invaginations with bacterial mesosomal-like structures that are rich in biosynthetic enzymes (Fig. 7), although we do not yet know which quorum-sensing controlled genes are involved in this process. For example, quorum-sensing controlled type VI secretion [16], whose multi-component machine is implicated in biofilm formation [43] and related biofilm-specific antibiotic resistance [44].

Thus, it is possible that the intracellular mesosomes [42], the surface's invaginations (present work), and the outer membrane vesicles [45]–[47] are simultaneous manifestations of one phenomenon of bacterial cell differentiation associated with QS-controlled biofilm development

Future investigation of this mechanism could reveal insights into both the function of quorum sensing in biofilm formation and the features of bacterial cell morphological differentiation associated with biofilm development and directed by quorum-sensing autoinducers.

## Supporting Information

Figure S1 AFM images (height signal) of *C. violaceum* NCTC 13274 growing for 24 (a, d), 48 (b, e) and 72 h (c, f) in planktonic conditions without (a–c) and with 0.1 µM C_6_-HSL (d–f). Scale bar – 1 µm.
(TIF)
