# Supplementary figures and images for: Atomic Force Microscopy Reveals a Morphological Differentiation of Chromobacterium violaceum Cells Associated with Biofilm Development and Directed by N-Hexanoyl-L-Homoserine Lactone
Source: PLoS One. 2014 Aug 11;9(8):e103741. doi: 10.1371/journal.pone.0103741 (PMC4128650; doi:10.1371/journal.pone.0103741)

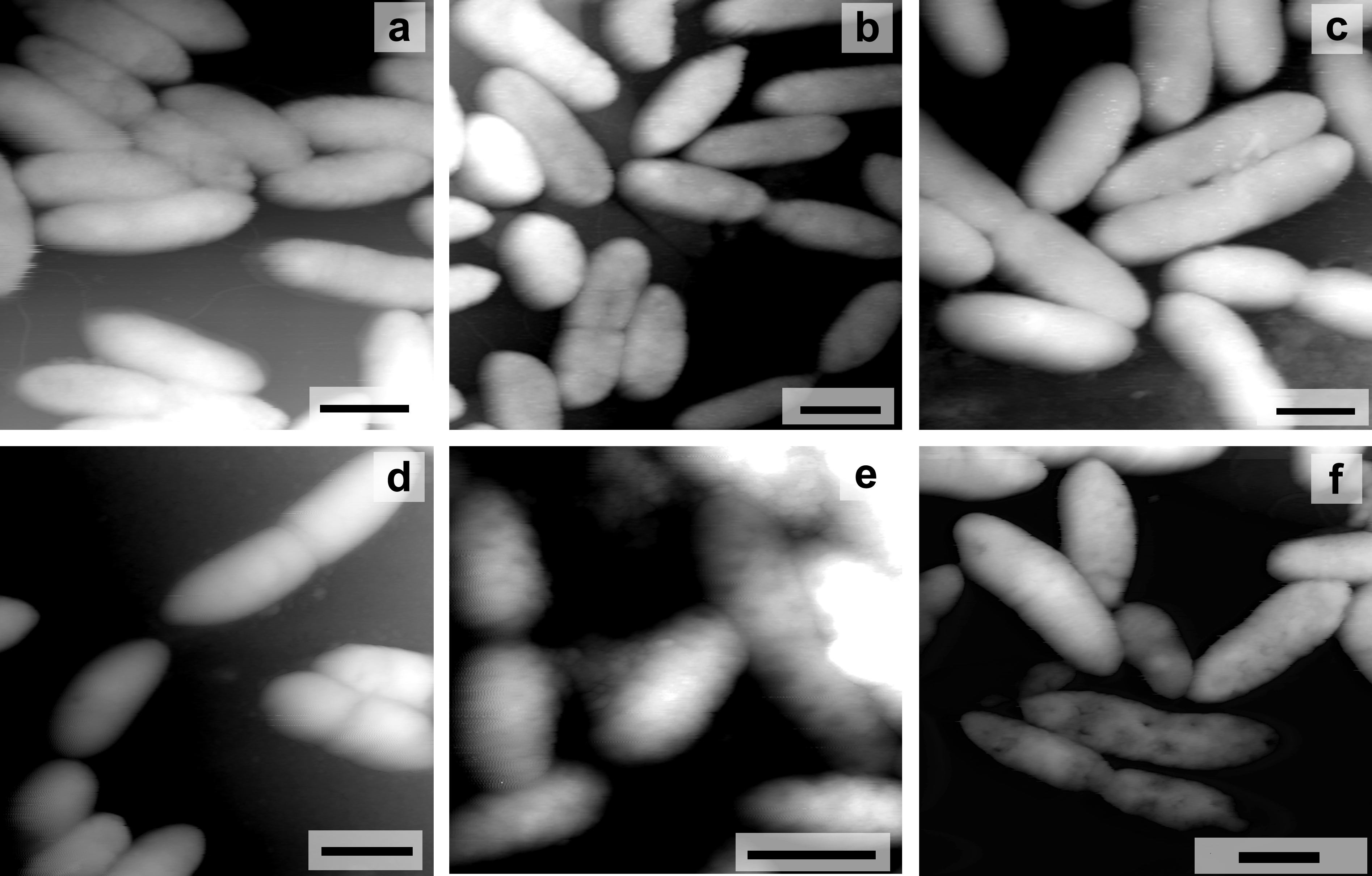

Supplement: Figure S1 — AFM images (height signal) of C. violaceum NCTC 13274 growing for 24 (a, d), 48 (b, e) and 72 h (c, f) in planktonic conditions without (a–c) and with 0.1 µM C6-HSL (d–f). Scale bar – 1 µm. (TIF) [file pone.0103741.s001.tif]
